# Femoral Neck Osteotomy: A Salvage Procedure for Unstable and Locked Acetabulum Fractures in Selected Frail Patients

**DOI:** 10.1007/s43465-021-00584-2

**Published:** 2021-12-16

**Authors:** Charlotte Cibura, Emre Yilmaz, Dina Straeter, Thomas A. Schildhauer, Christiane Kruppa

**Affiliations:** 1 Department of General and Trauma Surgery, BG-University Hospital Bergmannsheil, Ruhr-University, Bochum, Germany; 2 Chirurgische Klinik und Poliklinik, BG-Universitätsklinikum Bergmannsheil Bochum, Ruhr-Universität Bochum, Bürkle-de-la-Camp Platz 1, 44789 Bochum, Germany

**Keywords:** Geriatric patients, Elderly, Acetabular fractures, Femoral neck osteotomy, Multimorbidity, Salvage procedure, Locked femoral head, Locked geriatric acetabular fractures in selected frail patients, Salvage solution, Femoral neck osteotomy, Discussion of this treatment concept based on 9 cases

## Abstract

**Introduction:**

Purpose of the study is to present and discuss the femoral neck osteotomy as a salvage procedure for unstable, locked geriatric acetabular fractures in selected frail patients. When disadvantages and possible risks of other treatments exceed the benefits, this method may relieve pain and allow for early wheelchair mobilization in frail patients with limited mobility.

**Materials and Methods:**

We report nine patients from 2008 to 2020, which were treated with an osteotomy of the femoral neck for an unstable acetabular fracture. Indications, ASA-Score, Frailty Index, operative procedure, length of hospital stay, complications and outcomes will be discussed.

**Results:**

Patient’s age averaged 86 years (range 81–92). Acetabular fractures were classified as six both column fractures, two anterior column posterior hemitransversal fractures and one destruction of the acetabulum by multiple metastases. Fracture dislocation with medialization plus locking of the femoral head and a superomedial dome impaction were present in all patients. All patients were classified as ASA III/ IV and the average value on the CSHA Frailty index was 7 (range 6–7). The operation time averaged 52 min (range 34–62). Immediate wheelchair mobilization in seven out of nine patients was started postoperatively.

**Conclusion:**

The osteotomy of the femoral neck may be discussed as a salvage procedure in low functional demanding, multimorbid, frail geriatric patients with unstable acetabular fractures and impairment of mobilisation due to a locked femoral head. The procedure has the advantages of a short operation time and immediate mobilization of the patients. However, this procedure only applies as a salvage solution in selected individual cases.

## Introduction

Due to demographic changes, an increasing population age can be observed worldwide [1, 2]. In addition, an increasing number of acetabular fractures can be observed in geriatric patients. Ferguson et al. reported a 2.4-fold increased number of acetabular fractures in patients over the age of 60 years during the second half of the period between 1980 and 2007, compared to the first half of this period. Furthermore, patients over the age of 60 years had an increased amount of all acetabular fractures [3]. Although some high-energy trauma in fit geriatric patients are reported, most patients suffer their injury from low energy traumata, such as a fall from the standing position [4, 5]. The fractures are often characterized by a combination of an anterior column or wall fracture and fracture of the quadrilateral plate, which results in medialization and subsequent locking and destruction of the femoral head. In cases of an additional osteopenia two column acetabular fractures are likewise present [6]. No consensus regarding the optimal treatment method in this patient group exists and advantages and disadvantages concerning plate fixation, primary or secondary hip arthroplasty or non-operative treatment of unstable acetabular fractures are discussed [7–12]. The majority of geriatric acetabular fractures may be treated with one of the mentioned procedures, but for a small group of patients, who are not candidates for extensive surgery due to an extensively limited functional status, considerable dementia or a malignoma in palliative situation, with limited expected lifetime in combination with multiple significant comorbidities, other treatment options might be considered. This is particularly the case if femoral rotation might be painfully blocked by locking of the femoral head within the fracture or by extensive medialization of the femoral head, which results in further immobilization of the patient and difficulties in patient care. Especially since Anglen et al. have already described that osteopenic geriatric patients with superomedial dome impaction (Gull sign) did not benefit from attempted open reduction and internal fixation [13]. For those patients an osteotomy of the femoral head as a salvage procedure with a functional girdlestone situation should be discussed to reduce pain, to avoid a femoral traction and allow for early and free mobilisation of the hip and avoid further immobilisation.

The purpose of this study was to analyse and discuss the femoral neck osteotomy as a salvage procedure in individual multimorbid frail/bedridden geriatric patients with unstable and locked acetabular fractures.

## Materials and Methods

This is a retrospective study over a period of 12 years in a level 1 trauma center. Ethical permission for this study was obtained from the institutional ethical committee. Patients were identified using the OPS (operative procedure)—code 5–781.ae. All patients, who underwent an osteotomy of the femoral neck for an acetabular fracture during January 2008 and July 2020, were included. Patients with additional plate osteosynthesis in the area of the acetabulum during the same inpatient stay were excluded.

A total of 9 patients, four women and five men underwent the procedure. Using the patient’s hospital charts, we retrospectively recorded the demographic data, associated injuries, pre-existing comorbidities, the CSHA Frailty Index and the preoperative physical status according to the American Society Anesthesiologists (ASA-PS) classification system (Table 1) [14, 15]. The plain radiographs and computertomographic (CT) scans were evaluated and acetabular fractures were classified according to Judet and Letournel [16]. Furthermore, all CT scans and native X-rays were checked based on Anglen et al. and Butterwick et al. with regard to the following possible negative outcome parameters for osteosynthesis: "gull sign" (superomedial dome impaction), posterior wall comminution, marginal impaction of the acetabulum, femoral head impaction fracture and medialisation of the femoral head [13, 17]. In addition, it was checked whether there was a locked femoral head and/or an engagement of the greater trochanter with the pelvis due to femoral head medialization. Operative treatment protocols and anaesthesiologic protocols were evaluated regarding the operative procedure, length of the operation, intraoperative blood loss, anaesthesia and intraoperative complications. The length of hospital stay, postoperative mobilization, pain medication and postoperative complications, such as wound infections, bleeding and neurological or thromboembolical disorders were further evaluated.

**Table 1:** ASA- PS classification system

ASA	Definition	Examples, including but not limited to:
I	Normal healthy patient	Healthy, non-smoking, no or minimal alcohol use
II	Patient with mild systemic disease	Mild diseases only without substantive functional limitations. Current smoker, social alcohol drinker, pregnancy, obesity (30 < BMI < 40), well- controlled DM/HTN, mild lung disease
III	A patient with severe systemic disease	Substantive functional limitations; One or more moderate to severe diseases. Poorly controlled DM or HTN, COPD, morbid obesity (BMI > 40), active hepatitis, alcohol dependence or abuse, implanted pacemaker, moderate reduction of ejection fraction, ESRD undergoing regularly dialysis, history (< 3 months) of MI, CVA, TIA or CHD/stents
IV	A patient with severe systemic disease that is a constant threat to life	Recent (< 3 months) MI, CVA, TIA or CHD/stents, ongoing cardiac ischemia or severe valve dysfunction, severe reduction of ejection fraction, shock, sepsis, DIC, ARD or ESRD not undergoing scheduled regularly dialysis
V	A moribund patient who is not expected to survive without the operation	Ruptured abdominal/thoracic aneurysm, massive trauma intracranial bleed with mass effect, ischemic bowel in the face of significant cardiac pathology or multiple organ/system dysfunction
VI	A declared brain-dead patient whose organs are being removed for donor purposes	

*DM* diabetes mellitus, *HTN* hypertension, *COPD* chronic obstructive pulmonary disease, *ESRD* end stage renal disease, *MI* Myocardial infarction, *CVA* cerebro-vascular accident, *TIA* transient ischaemic attack, *CHD* coronary heart disease, *DIC* disseminated intravascular coagulation, *ARD* acute renal disease

### Operative Treatment

**Fig. 1:**
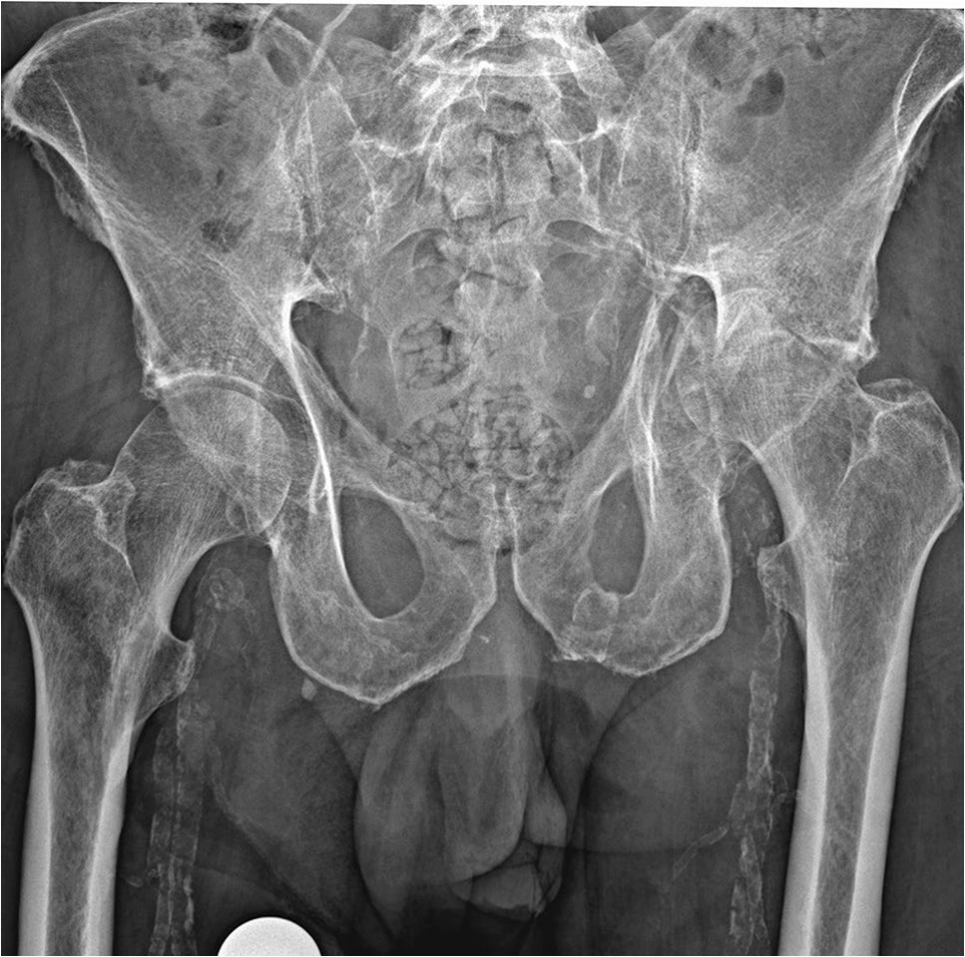
81-Year-old male (ID 7) preoperative anterior–posterior view with a both column acetabular fracture

**Fig. 2:**
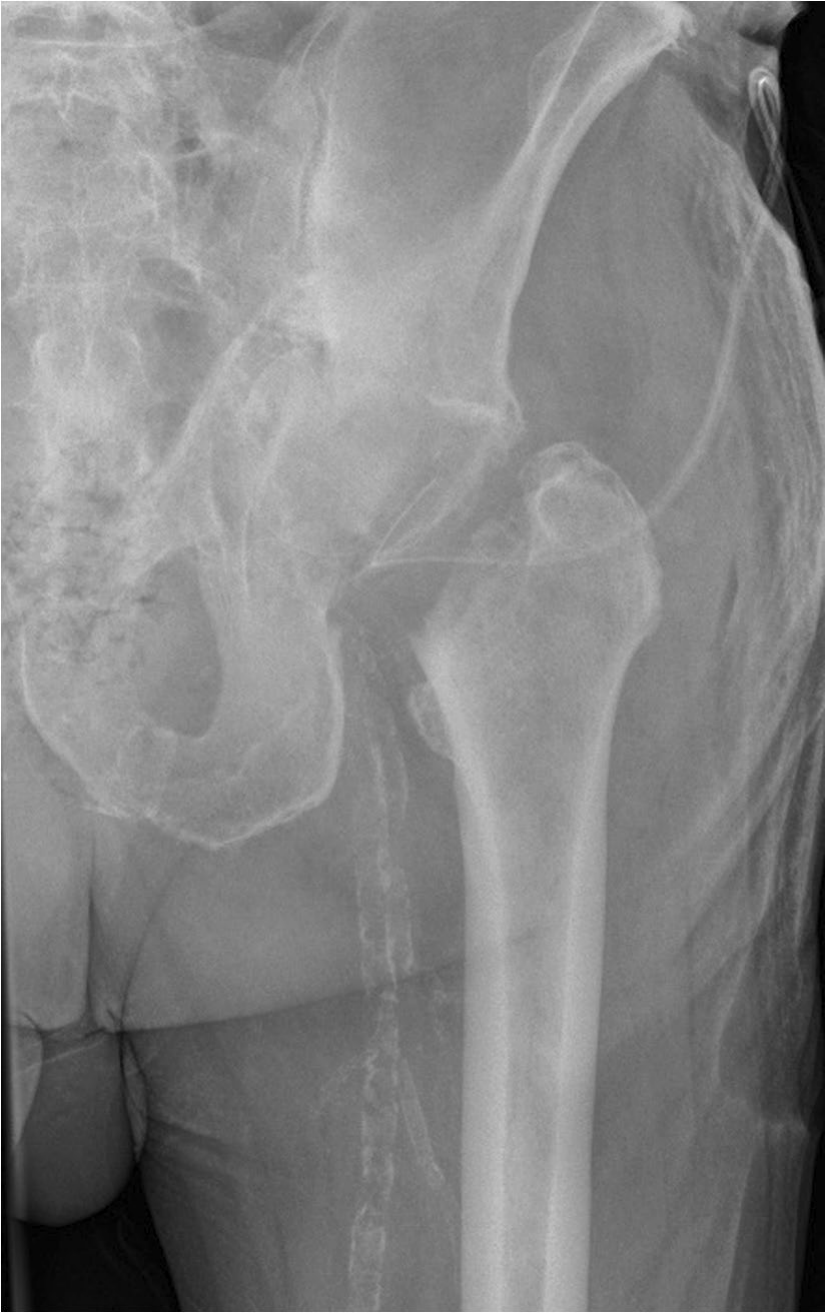
Postoperative anterior–posterior view after femoral neck osteotomy

All patients were placed in a supine position. A single shot antibiotic infusion (2 g Cefazolin) was administered preoperatively. Either a Bauer or Watson–Jones approach was used. The hip capsule was visualized, T-shaped incised and suture marked. The femoral neck was secured by two round Hohmann hooks and the hip externally rotated. Using an oscillating saw two osteotomies of the femoral neck were made to produce an approximately 2-cm-wide slice of the neck, which was then removed. Afterwards, free rotation of the leg without contact within the osteotomy zone was ensured visually. The femoral head remained in situ (Figs. 1, 2). After irrigation of the wound, the hip capsula was sutured and a drain inserted. The wound was subsequently closed. Daily wound controls followed. The drain was removed on the second or third postoperative day, as soon as < 50 ml/day drained out. Patients were allowed to mobilize with toe touch weight bearing in a standing position, sitting position in a wheelchair or in the patient’s bed, depending on their general health status and pre-existing mobilization status.

## Results

The patient’s age averaged 86 years (± 4.8; 81–92 years). All patients had multiple comorbidities (Table 2) and all patients had one or more diseases from the following categories: neurological disorders (Parkinson's disease, dementia etc.), severe cardiac diseases (heart failure, post-myocardial infarction, coronary heart disease, etc.) and/or underlying malignant disease (multiple myeloma, breast cancer etc.). Six patients were living in a nursing home due to their previous illnesses/overall physical condition and six patients could not consent to the operation on their own. The patients were preoperatively divided according to the ASA-PS classification system as follows: Five patients ASA III and four patients ASA IV [14]. The average value on the CSHA Frailty index was 7 (range 6–7) [15]. Six of the patients were no longer mobile independently and were only mobilized in the wheelchair, the other patients were only mobile a few steps or short distances on the rollator. The mechanism of injury was in all patients a fall from a standing or sitting position (low-energy trauma). Acetabular fractures were classified as follows: six both column fractures, two anterior column posterior hemi-transversal fractures and one destruction of the acetabulum by multiple metastases. All patients had a positive “Gull sign” as a negative outcome parameter and all had a fracture dislocation with medialization and locking of the femoral head (Table 3). One patient had an associated humeral head fracture and one a spine fracture. No secondary congruence was found in any of the two column fractures. The average time to the osteotomy of the femoral neck was 5 days (± 5.5; 0–14 days). In two patients, the operative treatment was delayed because of preoperatively performed tumor staging and tumor board discussion and necessity of legal support implementation. One patient required intensive care treatment at the time of admission because of a reduced health status. In five patients, a femoral traction was applied during admission and remained until the operative treatment. In all patients, only an osteotomy of the femoral neck was performed. The operation time averaged 52 min (± 10.8; 34–62 min). The operation was conducted under intubation anaesthesia in six patients and under laryngeal mask in combination with regional anaesthesia in three patients. The average number of packages of red blood cells was 1.5 (0–6), which were given perioperatively, four patients required no transfusion. Four patients were permanently anticoagulated preoperatively. The documented patient’s blood loss was less than 300 ml intraoperatively in all patients. Two patients required blood transfusions postoperatively, because of a drop down of their haemoglobin level without necessity for operative revision. One patient had symptoms of a transient ischaemic insult with anisocoria and dysarthria without any findings on the brain computer tomographic scan. All symptoms resolved completely. Two patients showed a postoperative delirium and required admission to an intermediate care unit postoperatively. One patient developed a nosocomial pneumonia, which was treated with antibiotics. Four patients were admitted to an intensive care unit postoperatively. One patient was admitted to an intensive care unit with admission because of the reduced mental health and comorbidities. The length of hospital stay averaged 15,3 days (± 6.7; 6–25 days). The patient who had been admitted to the intensive care unit preoperatively deceased 13 days after surgery because of a septic multiorgan failure due to pneumonia and a DeMars catheter infection. Another patient deceased 22 days postoperatively in an in-patient palliative care unit, because of severe dementia and an ischaemic insult with hemiplegia. Patients were mobilized in a sitting position at postoperative day one, depending on the individual health status. Wound infections were not observed and no operative revisions were necessary. Pain medication at discharge was in seven patients 4 × 500–1000 mg Novaminsulfon/day, in one case in combination with a transdermal Buprenorphine application, which was already part of the patient’s previous medication. One patient was discharged with his preadmission medication only (transdermal Buprenorphine). Two patients were discharged to a nursery home, one had 24/7 nursery at home, four patients were discharged into a geriatric early complex care rehabilitation and one patient was referred to an in-patient palliative treatment. In two of the patients, based on the final report of the geriatrics one month after surgery, mobilization in the wheelchair could be recorded once with help and once without help while maintaining pain medication. One of these patients was able to stand on the walking frame with little help and was discharged home with a nursing service. In the other patient, the transfer was only possible with help, but this was due to dementia and hypoactive delirium. A third patient with severe dementia deceased 5 months after surgery for unknown reasons. According to the relatives, a transfer to the wheelchair by nursing staff was possible but with increased pain medication.

**Table 2:** Demographic data and pre-existing comorbidities

ID	Age (years)	Pre-existing comorbidities	Hemo-dialysis	Cardiac pacemaker	Malignant tumor	Dementia
1	84	Parkinson’s disease, DM (type II), lymphadenectomy, Ileal conduit, post-radiation	–	–	+ (metast. bladder Ca, metast. Prostate Ca)	–
2	92	Vertebrobasillary TIA, cerebral artery media stenosis (l), chronic subdural hematoma, heart failure, post MI, CHD, sick sinus syndrome, atrial fibrillation, DM (type II)	–	+	+ (Mamma Ca, mastectomy)	+
3	92	Cardiorenal syndrome type 2, heart failure NYHA IV, dilatative cardio-myopathy, mitral III°, aortic II-III° regurgitation, a-v block III°, HTN, hypothyroidism, restless leg syndrome	+	+	–	–
4	87	Exsiccosis, deterioration of general health, anemia	–	–	–	+
5	84	DM (type II), PAOD, pneumonia, atrial fibrillation	–	–	–	–
6	81	DM (type II), ESRD, HTN, CHD, artrial fibrillation,TIA, esophageal varices with previous upper gastrointestinal bleeding	+	–	+ (Multiple myeloma)	–
7	81	Pneumonia, Parkinson’s disease, DM (type II), CHD, cardiac stenting, atrial fibrillation, mitral and aortic regurgitation, history of Poliomyelitis, carotid stenosis, HTN	–	–	–	+
8	91	CVA with hemiplegia, osteoporosis, delirium, depressions, suicide attempt	-	-	-	+
9	90	TIA, macula degeneration, presbycusis, HTN	–	–	–	+

*Ca *carcinoma, *metast. *metastasized,  +  yes, —no, *PAOD* peripheral artery occlusive disease, *COPD *chronic obstructive pulmonary disease, deterioration of general health, *DM *diabetes mellitus, *CHD *coronary heart disease, *TIA *transient ischaemic attack, *l *left, *sec*. secondary, *A. *arterial, *MI *myocardial infarction, *HTN *hypertension, *ESRD *end-stage renal disease, *CVA *cerebro-vascular accident

**Table 3:** ASA-PS Score, Frailty Index and fracture classification

ID	ASA	Frailty- index	Fracture type	Gull sign	Femoral head medialization	Locked femoral head	Engagement of the greater trochanter	Femoral head impaction fracture	Comminution of posterior wall fractures	Marginal impaction associated with posterior wall fractures
1	III	7	Osteolysis (malignoma)	YES	YES	YES	NO	NO	NO	NO
2	IV	7	Ant. Column + post.hemitr	YES	YES	YES	YES	NO	YES	YES
3	IV	7	Both columns	YES	YES	YES	NO	NO	YES	YES
4	III	7	Both columns	YES	YES	YES	NO	YES	YES	YES
5	III	6	Both columns	YES	YES	YES	NO	NO	YES	YES
6	IV	7	Both columns	YES	YES	YES	YES	NO	NO	YES
7	IV	7	Both columns	YES	YES	YES	YES	YES	YES	YES
8	III	7	Both columns	YES	YES	YES	YES	NO	NO	YES
9	III	7	Ant. Column + post.hemitr	YES	YES	YES	NO	YES	YES	YES

## Discussion

Despite the treatment goals in young patients with acetabular fractures, which are prevention of posttraumatic arthritis, preservation of the femoral head and achievement of a high functional outcome, treatment goals in geriatric patients are different. This is especially true in those, who require permanent care, who may be bedridden, who have multiple severe comorbidities and a reduced health and mobility status. This patient group requires differentiated and individually adapted treatment concepts. To relief pain, restore and allow early mobility and to prevent further fracture and immobility associated complications, should be the primary goals in this patient group. Different treatment concepts are discussed for these patients, such as non-operative treatment, percutaneous fixation, open reduction and internal fixation, primary hip arthroplasty with or without additional plate fixation [10, 18–20].

The purpose of this study was to introduce and discuss the osteotomy of the femoral neck in this context as a salvage procedure focussing on selected frail patients with clearly reduced functional and general health status due to dementia, malignoma and/or multimorbidity. All presented patients suffered from an unstable acetabular fracture and would have completed the criteria for operative fixation with the typical characteristics of a geriatric acetabular fracture. Firoozabadi et al. characterized the typical acetabular fracture pattern in geriatric patients as a low anterior column component in combination with a posterior column fragment with a lager quadrilateral plate defect which is cranio-medially displaced [21]. Also Ferguson et al. reported the separated quadrilateral plate as a typical geriatric fracture component [3]. The Gull sign was described by Anglen et al. as a negative outcome parameter that predicted patients in whom adequate reduction and stable fixation could not be obtained. It is described as the impaction of a portion of the subchondral bone of the anteromedial roof into the osteopenic supporting bone [13]. Laflamme et al. confirmed this and in further studies further factors for a poor outcome were described which are summarized again in a study by Butterwick et al. [6, 17, 22]. All the patients in this study also showed these factors with a clear medialization of the femoral head so that one has to assume a insufficient result after osteosynthesis. Furthermore all patients had multiple pre-existing comorbidities with a noticeably high Frailty Index. Due to this, the general health status was reduced and the perioperative risk increased, which prohibited extensive operative procedures, especially since no good operative outcome was to be expected. In this situation, the osteotomy of the femoral neck has the advantages of a relatively small operative procedure with a short operation time, which in our patients averaged 52 min, and low intraoperative blood loss. Further medialization of the femoral head under weight-bearing and painful blocking phenomenon with femoral head rotation can be avoided. Early mobilization, in terms of mobilization from bed, into a sitting position, wheelchair or even in a standing position are possible. Full weight bearing can be allowed after wound healing, but requires a heightening of the patient’s shoe. Sharma et al. could show in a study in geriatric patients with Girdlestone situation due to a failure after total hip prosthesis that a pain reduction could be achieved in 12 out of 14 patients and 10 patients were able to walk with aids [23]. But, the hospital course of the present patients illustrates, that even with this relatively small procedure, complications such as pneumonia and neurological disorders occur, the hospital stay may be lengthen by them and intensive care medicine might be necessary. In the present patient, the average time until the operation of six days was also responsible for the relatively long hospital stay, which resulted from preoperatively required examinations, legal authority arrangements and decisions making process with patients and relatives as well as the necessity of preoperative intensive care treatment. It would be desirable to shorten the preoperative time, but from our experience, the reported problems are often present in geriatric patients. However, for the reported cases it is certainly worth to discuss, whether a non-operative treatment of the unstable acetabular fracture would have been an alternative or not. Ryan et al. described in 26 non-operatively treated patients (mean age 76 years) with unstable acetabular fractures, that the majority of patients did not have significant functional deficits and pain at 2 years after their injury [20]. But it remains unanswered how long the patients were limited in their mobility after the injury because of pain. Five patients were treated with a femoral traction at admission until the operative intervention. Continuation of the traction until fracture healing should be discussed as another treatment option. Indeed, this results in further immobilisation and hospitalization in patients with an already decreased health status. Firoozabadi et al. treated 16 geriatric patients with unstable acetabular fractures non-operatively with femoral traction, because of their reduced health status. The traction remained for 4 to 6 weeks. The 1-year-mortality rate in these patients was 79%, of which 47% died within the first 90 days. In comparison, the 1-year-mortality rate of 77 patients, who had a stable acetabular fracture and were non-operatively treated without femoral traction was only 44% [21]. This underlines the assumption, that in fracture types, which would require a femoral traction, the femoral neck osteotomy should be discussed as an alternative salvage procedure to enable early mobilisation. Further, it should be questioned, if, when the patients will be operatively treated, this should not be a fracture fixation or an arthroplasty. But, both procedures are challenging, especially in this patient population, even in the hands of experienced surgeons, despite modified operative approaches or fracture bridging arthroplasties. The operation usually requires more time and results in higher blood loss. Regarding plate fixation the question needs to be answered, if there really is a benefit for the patient. The patient does not benefit from an earlier weight bearing compared to the osteotomy, having the usually decreased bone mineral density in mind, which allows no weight bearing until the fracture is healed. However, Firoozabadi et al. were able to determine a clearly lower 1-year-mortality of 12% in their study compared to those who received no surgical treatment [21]. The total hip arthroplasty in contrast might be able to allow early weight bearing in theory, but should not be compared to primary hip arthroplasty in patients with a coxarthrosis. Rickmann et al. showed in 24 patients (average age 77 years) that immediate weight bearing as tolerated is possible after total hip arthroplasty for acetabular fractures without secondary dislocation, but in all patients additionally plate fixation of both acetabular columns were performed and a tantal-augmented prosthesis has been used resulting in an operation time of 193 min and an average blood loss of 1100 ml [18]. The 1-year-mortality here was 14% and Daurka et al. could not determine any significant difference in mortality according to ORIF (open reduction and internal fixation) and ORIF + total hip arthroplasty in those over 55 years of age [11, 18].

But, all three versions enable, as well as the femoral neck osteotomy, early mobilisation in bedridden patients, mobilization in a sitting position or wheelchair. The individual benefit needs to be critically evaluated for each patient.

In our opinion, the algorithm to treat geriatric patients with acetabular fractures according to Mears et al. should be expanded with the femoral neck osteotomy as a treatment option [6]. In the present patients with reduced health status and dementia it is difficult to objectify the clinical outcome. Therefore, we are only able to draw indirect conclusion regarding our primary goal to reduce pain and allow early mobilization. Seven patients were wheelchair mobilized at the date of discharge and in the course of the geriatrics, one patient was able to be mobilized to stand with little help on the walker. The only pain medication that was given additionally to the patient’s home previous medication was Novaminsulfon and in one patient additionally Tilidine after discharge. Therefore, the functional outcome may be assumed as improved compared to preoperatively when it had been unable to mobilize the patients because of pain.

## Limitations

Limitations are present due to the small patient group, which does not allow to-draw general conclusions for all patients. Further, the described procedure is a salvage procedure and should remain as one. It should not be misunderstood to be a competing procedure. Our goal to relief pain and allow early mobilization was not compared to other operative or non-operative procedures in this article. Therefore, we can only draw valid conclusions from the documented pain medication and mobilization at the date of discharge.

## Conclusions

Treatment of acetabular fractures in geriatric, multimorbid and frail patients is challenging. Besides the options of non-operative treatment, percutaneous fixation, plate fixation, arthroplasty or combinations of them. The femoral neck osteotomy should be discussed as a salvage procedure in selected patients, especially in functionally low demanding patients with increased perioperative risk, when the femoral head is widely medialized and further medialization and locking phenomenon are present or imminent with mobilization. It is not intended to represent a generalized treatment method for geriatric patients, but should only be discussed in special individual cases (as shown here) to enable quick, less painful mobilization with a much smaller intervention.
